# Ignored Faces Produce Figural Face Aftereffects

**DOI:** 10.1371/journal.pone.0045928

**Published:** 2012-09-21

**Authors:** Janice E. Murray, Madeline Judge, Yan Chen

**Affiliations:** Department of Psychology, University of Otago, Dunedin, New Zealand; University of British Columbia, Canada

## Abstract

Following adaptation to faces with contracted (or expanded) internal features, faces previously perceived as normal appear distorted in the opposite direction. This figural face aftereffect suggests face-coding mechanisms adapt to changes in the spatial relations of features and/or the global structure of faces. Here, we investigated whether the figural aftereffect requires spatial attention. Participants ignored a distorted adapting face and performed a highly demanding letter-count task. Before and after adaptation, participants rated the normality of morphed distorted faces ranging from 50% contracted through undistorted to 50% expanded. A robust aftereffect was observed. These results suggest that the figural face aftereffect can occur in the absence of spatial attention, even when the attentional demands of the relevant task are high.

## Introduction

Faces are arguably one of the most salient classes of stimuli we encounter in the visual environment, yielding crucial information about gender, identity, race and emotional state. It has been proposed that faces are represented in some multi-dimensional face space centered on a norm or average face. The norm represents the average values of the dimensions that are used to discriminate among faces, and individual faces are located in face space relative to the norm (norm-based coding model, [1]). On a daily basis we typically experience many faces, both unfamiliar and known. This repeated exposure to new and varied faces requires our internal representation of faces (e.g., the norm) to be modified if we are to effectively extract and interpret the important information that faces afford. Research using adaptation techniques suggests that, remarkably, we can update the norm with limited exposure to a face (e.g., [2], [3]). For example, after briefly viewing a face with its internal features expanded away from the centre of the face, we perceive an undistorted face as having contracted facial features, and the face that is most normal-looking shifts toward the adapting distortion [3]. In addition to figural face aftereffects, identity-, emotion-, race-, and gender-specific aftereffects also occur following adaptation to faces [2], [4]–[6], and these aftereffects suggest very responsive processing mechanisms are constantly at play to provide the most up-to-date internal representation of faces.

A recent line of inquiry has explored possible boundary conditions for face aftereffects with mixed results. Moradi et al. [7] reported that when the adapting stimulus is rendered invisible through interocular suppression (binocular suppression or continuous flash suppression), adaptation to face identity does not occur, leading the authors to conclude that face aftereffects depend on conscious perception of the adapting face. Gender-specific adaptation also appears not to occur when faces are fully suppressed [8], [9], and neither is there strong evidence for adaptation to race [8] or face shape [10]. On the other hand, adaptation to emotional facial expression is reduced but not always eliminated when awareness of the adapting faces is absent due to suppression [11], [12]. Finally, Moradi et al. [7] paired low-contrast adapting faces with a demanding working-memory task to create conditions of inattentional blindness. Under these conditions, the participants who were purportedly unaware of the adapting faces showed no significant face identity aftereffect (it is of note that the null effect was obtained with a small sample size of seven and approached significance).

Experiments using interocular suppression techniques are primarily concerned with the fate of suppressed stimuli and the role that awareness plays in face aftereffects. In our experiment we took a different approach to understanding face aftereffects and considered the possible influence of visible faces that we are aware of, but do not attend to. In a complex visual environment, selective attention mechanisms operate to prioritize stimuli for processing to prevent overload of our cognitive system (e.g., [13]), selecting some information to be processed with the remainder ignored. Because of their sociobiological significance, faces have been considered a likely class of stimuli to operate outside the constraints of selective attention. Finkbeiner & Palermo [14] have reported that the nonconscious processing of gender occurs without attention. Also, unlike other classes of stimuli, the identity of task-irrelevant faces seemingly can be processed irrespective of the difficulty or perceptual/attentional load associated with the task-relevant stimulus [15], [16], prompting the conclusion that face processing may occur in the near absence of attention [17]. However, other work indicates that not all aspects of unattended faces are processed, particularly when the attentional demands of the relevant task are high. The race and gender of ignored faces is processed under conditions of low but not high attentional load [18], and attentional load may also play a role in processing fearful facial expressions [19] and threat-relevant faces [20]. Thus, there is no definitive answer to the question of the processing fate of ignored faces, with the varied results suggesting that several variables, including type of task and task demands, are relevant in developing an understanding of whether face processing occurs independent of attentional resources.

The studies of awareness and adaptation effects discussed above do not bear directly on our question of whether figural aftereffects occur in the absence of spatial attention. In suppression tasks, participants are typically asked to monitor their perception of the suppressed stimulus and report when it is perceived, and for this reason, the adapting stimuli in these tasks cannot be considered unattended or task-irrelevant. To address the novel question of whether adaptation occurs when *participants are aware of, but ignore, task-irrelevant faces*, we used the figural aftereffect paradigm and asked participants to perform a highly demanding relevant task in the presence of an unattended, visible adaptation face. Participants were required to count the number of briefly presented red Ts in a rapid serial visual presentation (RSVP) stream of red and blue Xs and Ts while ignoring an adapting face simultaneously presented to the left or right of the RSVP stream. Importantly, adapting faces and their location were never task relevant. In addition to presenting an attention-demanding task, we further maximized the likelihood that spatial attention was strongly directed toward the relevant task by offering a monetary incentive linked to task performance. To foreshadow our results, we found that when attention is directed away from task-irrelevant adapting faces, figural aftereffects do occur even when the attentional load in the relevant task is high.

## Method

### Ethics Statement

This research was approved by the University of Otago Human Ethics Committee, and all participants provided written informed consent prior to participation.

### Participants

Twenty Caucasian University of Otago students (13 females) aged 18 to 29 years participated in the experiment for a payment of $12. All participants had normal or corrected-to-normal vision.

### Stimuli

**Figure 1:**
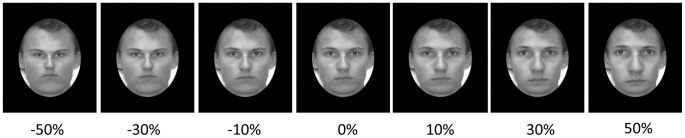
Representative examples of the 11 distorted test faces rated for normality. Percent distortion is shown below each image.

Greyscale photos of eighteen Caucasian male faces presented in frontal view with neutral expressions served as the test and adapting stimuli. Each face was set in an oval surrounded by a black rectangle that obscured any significant hair and ear features. Ten of the faces were used to create the 110 test stimuli for the pre- and post-adaptation phases of the experiment. The internal features of each of these test faces were expanded or contracted in 10% steps from 0% to+or –50% distortion to yield 11 images per face (see Figure 1 for examples. The individual in Figure 1 has given written informed consent to use his photo in scientific publications.). The test faces measured 9.6° by 13.4°. The remaining eight faces, with internal features contracted 50%, were used as adapting faces and measured 4.8° by 6.8°. The difference in size (and spatial location, see below) of the adapting and test faces insured that any aftereffects would implicate non-retinotopic face-coding mechanisms (e.g., [21], [22]). The letters “X” and “T” colored either blue or red (Arial font, size 28) were used as the attended stimuli in the adaptation phase. E-Prime [23] was used to present the displays, control timing of events and record responses.

### Design and Procedure

All participants experienced pre-adaptation, adaptation and post-adaptation phases. In the pre-adaptation phase, each of the 110 test faces was centrally presented for 1500 ms with the word 'rate' appearing above and below the face. Participants were instructed to view the face and then rate it for normality on a scale from 1 (unusual) to 9 (normal). With the offset of the test face, the rating scale was presented and remained on until the participant's response. Following the response, a blank screen was presented for 500 ms prior to the onset of the next trial.

In the adaptation phase that followed, an adapting face was presented 4.8° to the left or right of an RSVP stream centered at fixation. An adaptation trial began with a fixation cross displayed in the center of the screen. After 500 msec, the fixation cross was replaced by the RSVP stream. Each of the 16 letters in the RSVP stream was displayed for 235 ms, with a 15 ms inter-letter interval. The display times, established during pilot testing of the letter-count task in isolation, precluded saccades and provided for an appropriate level of task difficulty. Participants were instructed to report the number of red Ts in the RSVP stream and to ignore the face. The number of targets in any given trial ranged from zero to six. To help orient to the task and avoid missing a potential target at the beginning of the letter stream, a green X was always the first letter presented. The adaptation face was onset and offset simultaneously with the RSVP stream and thus displayed for 4.25 seconds on each trial. The assignment of red T targets to positions in the RSVP stream was random with the constraint that letters of the same color and identity were not permitted to appear consecutively. Therefore, the earliest that a second target could be presented was 500 ms after the presentation of a previous target, thus minimizing any potential attentional blink effects in the task [24]. The last letter in the RSVP sequence was followed by a “?” displayed for 500 ms to signal the response interval. The experimenter entered the participant’s verbal response using a numerical keypad on the keyboard. When an error was made, a tone was sounded for 500 ms to provide the participant with accuracy feedback. Over the course of 96 adaptation trials, each of the eight adapting faces randomly occurred an equal number of times to the left and right of the RSVP stream.

Finally, in the post-adaptation phase, participants were asked to rate the normality of the 110 test faces again. To maintain adaptation, each rating trial was preceded by a single adaptation trial. Participants provided their red T count in the 500 ms interval that immediately preceded the presentation of the test face to be rated. The experimenter entered the participant’s response following the rating trial, and accuracy feedback on error trials was provided before proceeding to the next adaptation/rating trial sequence. Each of the eight adapting faces was presented at least six times to the right and six times to the left. To complete the required 110 trials, adaptation faces on 14 trials were randomly chosen from the set of eight, with seven faces shown on the left and seven on the right.

To focus attention on the RSVP task during adaptation trials, participants were told that we were interested in how well they could count letters presented in rapid succession. To reinforce the importance of attending to the relevant task we further advised that $20 was being awarded to the individual who achieved the overall highest level of accuracy counting red Ts.

If face adaptation occurs in the absence or near absence of general attentional resources, then a significant figural aftereffect should be present when attention is focused on the highly demanding RSVP task and the task-irrelevant adapting faces are ignored; following exposure to unattended faces with contracted internal features, the most normal-looking face should shift toward the adapting distortion.

## Results

### RSVP Task

All twenty participants met an accuracy criterion of 70% on the letter-count task (an additional five participants who failed to meet the criterion were excluded). If attention was successfully directed to the letter count task, we expected task accuracy to be consistent with the benchmark level (86% correct) obtained in prior testing when a separate group of twenty participants performed the task without the adapting faces. This was the case. Mean percent correct in the letter-count task was 85% in both the adaptation and post-adaptation phases, and not significantly different from performance on the same task presented over the same number of trials in the absence of the adapting faces (*t* <1 in both phases).

### Normality Rating

**Figure 2:**
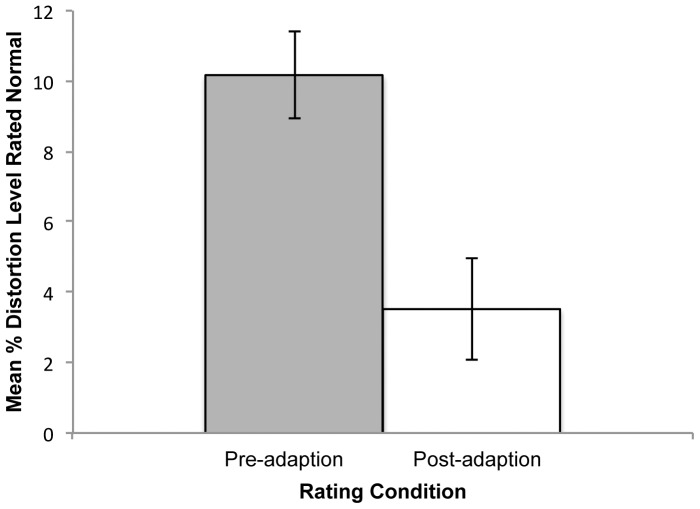
The mean percent disortion of the faces rated most normal is shown separately for faces rated prior to (Pre-adaptation) and after (Post-adaptation) adaptation. Standard error bars are given for each condition.

Participants’ normality ratings across the range of distorted faces from –50 to+50 were fitted with third-order polynomial functions, carried out separately for the pre- and post-adaptation phases. The fits of the polynomial functions to the individual data were excellent, with the mean *R^2^s* falling between.93 and.96. The peaks of the resulting functions for each participant were used to determine the distortion level that faces appeared most normal. The mean percent distortion levels for pre-and post-adaptation ratings are shown in Figure 2 (a preliminary analysis revealed that location of the adapting face, left or right of fixation, did not impact on distortion levels, and location was not considered further).

To determine any aftereffect, the mean percent distortion levels for the post-adaptation and pre-adaptation phases were compared. As evident in Figure 2, the distortion chosen as most normal shifted in the direction of the adapting stimulus in the post-adaptation phase as would be expected if exposure to the contracted adaptation face was effective in producing an aftereffect (e.g., [3]). A one-tailed *t*-test confirmed that the observed robust aftereffect was significant, *t* (19) = 4.30, *p*<.001.

## Discussion

In pairing a task-irrelevant adapting face with a highly demanding letter-count task, we sought to determine whether adaptation to global configural distortions of faces shifts what looks normal in the absence of spatial attention. Our results showed that indeed the perception of normality in test faces was shifted in the direction of the adapting distortion under conditions in which visible, irrelevant adapting faces were ignored. We are confident that the adapting faces were ignored given the brief presentation time of the individual letters in the relevant task, the high attentional load of the relevant task, and the monetary incentive that was effective in producing a level of performance that was virtually identical to performance of the relevant task in isolation. To the best of our knowledge, our results are the first to demonstrate that the figural face aftereffect can occur outside the focus of spatial attention even when the attentional demands of the relevant task are high.

Differences between the adapting and test faces in size and retinal position eliminated the contribution of low-level retinotopic mechanisms to the observed aftereffect [21], [22]. Thus, the figural aftereffects found here implicate post-retinotopic coding mechanisms. Encoding of the structural properties of a face (e.g., the configural relations among facial features) is central to our ability to recognize faces (for a review, see [25]), and it is plausible that the responsiveness to global configural distortions we observed reflects adaptation of high-level representations of whole-face structure. This further suggests that identity aftereffects would also be expected to occur when adapting faces are unattended. It is possible that mid-level, non-face-specific mechanisms that code general shape properties also contribute to the observed figural aftereffect [26], [27] but a variety of findings suggest that figural and identity face aftereffects at least partially signal adaption of higher-level, face-coding mechanisms (for a review, see [28]).

The results of our study are broadly consistent with the view that faces can be processed with minimal attentional resources [16], [17]. Given that the current study demonstrates that ignored faces produce robust aftereffects, it is relevant to consider the role attention plays in regulating mechanisms of face adaptation. One possibility is that attention might improve selectivity or enhance the adaptation processes resulting in stronger aftereffects (e.g., [29]). Recent work by Rhodes et al. [30] with attended adapting faces is consistent with the view that attention enhances adaptation to faces. In their studies, participants attended to visible adapting faces in a passive-viewing condition and, in enhanced-attention conditions. When attention to the adapting faces was enhanced, by requiring participants to either detect changes in brightness of lips or eyes or repetitions of adapting faces, the size of identity and figural aftereffects was increased. Previous findings with suppressed adapting faces also suggest attention may serve to modulate adaptation [9], [12]. In considering these findings together with the present results, we suggest that adaptive updating of norms occurs in the absence of attention, with the benefits of attention possibly manifested in increased coding efficiency and enhanced discrimination within the adapted population of faces [30].

The figural aftereffect in the present experiment resulted when a single distractor face was simultaneously presented with the non-face target. In our daily environment we typically encounter multiple stimuli, and one relevant consideration is the impact a cluttered environment has on processing of an unattended face. Jacques and Rossion [31], [32] showed that concurrent presentation of an attended face at fixation and an unattended lateralized face leads to a reduction in the N170 amplitude to the lateralized face relative to the amplitude observed when attending to a non-face stimulus or a scrambled face. Behavioral evidence reveals distractor congruency effects are diluted when the distractor face is accompanied by another face, but not by a meaningful object or inverted face [33] and can be eliminated with multiple distractor faces [34]. One possible explanation of these findings is that whereas faces are not subject to the limits of general processing resources [16] they may compete for limited, face-specific, resources [34]. If this is the case, the presence of numerous faces could alter the degree to which unattended faces yield adaptation effects.

Face gender and race also yield aftereffects [2], [4]–[6], and whether or not adaptation of these socially relevant dimensions occurs outside the focus of spatial attention warrants future consideration. Previous work reveals that gender and race aftereffects are bound to the perceptual categories comprising the dimension being examined [35]–[39]. Beselmeyer et al. [35], for example, observed opposite gender aftereffects for between-category faces (e.g., male, female), whereas no aftereffects were found for faces within one gender category (e.g., female, female) or the other. Notably, the structural differences between faces in the within-category group were mathematically identical to those in the between-category group. These findings implicate mechanisms that code perceptual category and it is plausible that such coding mechanisms have different attentional requirements. Murray et al. [18] found that the race and gender of distractor faces is processed in a flanker task when attentional load is low but not when it is high. Emotional expression is another important dimension of faces that may be processed in the near absence of attention (e.g., [40]), but like race and gender is sensitive to the attentional load of the accompanying relevant task [19], [41]. Thus, coding of category-related dimensions of faces may be more reliant on attentional resources, and if so, adaptation of these dimensions might not occur when the attentional demands of the relevant task are high.

Further studies will be needed to determine the precise mechanisms underlying the observed aftereffect with ignored adapting faces and the interplay between attention and the broader category of face aftereffects. Regardless of the outcome of future determinations, we can presently conclude that a visible face outside the focus of attention impacts on our subsequent perception of faces, renormalizing face space over the short term (at the very least). Accordingly, the face of the solitary unattended stranger that passes us on the street may have more influence on how we perceive faces than otherwise might have been expected.
